# A Method for Removing Amalgam Fillings

**Published:** 1868-06

**Authors:** 


*A Method for Removing Amalgam Fillings.*—Dr. J. Payne gives the following method for removing amalgam fillings which may save much time and labor: "I have two instruments made of silver, or silver points, somewhat resembling pluggers. One of these is straight, and strong enough to bear considerable pressure. The other is smaller, and bent at an angle of about forty-five degrees, so that it can be introduced between the back teeth. The points of both are flattened. When I wish to remove one or more plugs I scrape the surface bright and dip the silver-pointed iustrument into a quantity of quicksilver. The silver having an affinity for the mercury, a quantity will be taken up, adhering to the point which may be carried to the plug There is a stronger affinity between the quicksilver and the amalgam than between the quicksilver and the instrument, and it will therefore immediately leave the instrument and unite with the plug. The quicksilver will reduce the hardest amalgam plugs to their original plastic condition in a few minutes, when they can be removed without trouble. Ten or a dozen plugs may be removed in this way in as many minutes, which otherwise would be a good half day's work. The operation may be facilitated by using considerable pressure in rubbing the mercury on the plugs with the instrument. I rub it on all the plugs and then commence on the first one and cut into it with a strong ezcavator or sharp drill and then add more mercury and pass on to the next plug, and so on excavating and adding more mercury till I have gone over all the plugs. In the absence of the proper instruments, a strip of thick silver plate or a piece of silver wire fastened in any ordinary handle will answer as a substitute. Before dipping the point of the instrument in the mercury it should be well scraped to remove any oxidation caused by the mercury at a previous time."

